# Analytical Challenges and Regulatory Requirements for Nasal Drug Products in Europe and the U.S.

**DOI:** 10.3390/pharmaceutics6020195

**Published:** 2014-04-11

**Authors:** Sabrina Trows, Klaus Wuchner, Rene Spycher, Hartwig Steckel

**Affiliations:** 1 Department of Pharmaceutics and Biopharmaceutics, Kiel University, Grasweg 9a, 24118 Kiel, Germany; E-Mail: strows@pharmazie.uni-kiel.de; 2 Janssen Pharmaceuticals, Pharmaceutical Development & Manufacturing Sciences, Johnson & Johnson, Hochstrasse 201, 8205 Schaffhausen, Switzerland; E-Mails: kwuchne1@its.jnj.com (K.W.); rspycher@its.jnj.com (R.S.)

**Keywords:** nasal drug delivery, regulatory aspects, test methods, nasal sprays

## Abstract

Nasal drug delivery can be assessed by a variety of means and regulatory agencies, e.g., the Food and Drug Administration (FDA) and the European Medicines Agency (EMA) have published a set of guidelines and regulations proposing *in vitro* test methods for the characterization of nasal drug products. This article gives a summary of the FDA and EMA requirements regarding the determination of droplet size distribution (DSD), plume geometry, spray pattern and shot weights of solution nasal sprays and discusses the analytical challenges that can occur when performing these measurements. In order to support findings from the literature, studies were performed using a standard nasal spray pump and aqueous model formulations. The aim was to identify possible method-, device- and formulation-dependent influencing factors. The literature review, as well as the results from the studies show that DSD, plume geometry and spray pattern are influenced by, e.g., the viscosity of the solution, the design of the device and the actuation parameters, particularly the stroke length, actuation velocity and actuation force. The dominant factor influencing shot weights, however, is the adjustment of the actuation parameters, especially stroke length and actuation velocity. Consequently, for routine measurements assuring, e.g., the quality of a solution nasal spray or, for *in vitro* bioequivalence studies, the critical parameters, have to be identified and considered in method development in order to obtain reproducible and reliable results.

## 1. Introduction

The most prominent way of intranasal drug delivery is the administration of locally acting drugs in order to treat nasal congestion, infections and allergic rhinitis [1]. However, the nasal route can also be used for the systemic delivery of drugs for the therapy of various diseases, like osteoporosis and migraine, as well as for pain management and also for the administration of vaccines [2]. It is a painless, non-invasive delivery route, resulting in a rapid drug onset of action, due to the high vascularization of the nose and high permeability of the nasal mucosa under avoidance of first pass metabolism [3]. These advantages lead to high patient convenience and compliance.

For nasal drug delivery, there are several dosage forms available. The most popular examples are nasal sprays and nasal drops for which the drug can be formulated as a solution or suspension. Alternative dosage forms are the pressurized nasal aerosols and nasal powders. Typically, aqueous nasal spray formulations contain the drug, as well as bioadhesive polymers, surfactants, tonicity agents and, in some cases, penetration enhancers [4]. Bioadhesive polymers, like sodium carboxymethyl cellulose, are often used to increase the viscosity of the formulation in order to stabilize the suspension or to increase the residence time in the nasal cavity to modify drug absorption [4,5]. Surfactants can be included in the formulation to solubilize the drug in case of poor solubility or to increase the wettability [6].

Besides the formulation, also the delivery device plays an important role in nasal drug delivery, and only the combination of both, device and formulation, determines the properties of the final nasal drug product. This makes the development of nasal drug products more complex, since the variability of the formulation and the device have to be taken into account [5]. Therefore, the analytical requirements for the approval of nasal drug products exceed those for solid dosage forms [7]. For the *in vitro* characterization of nasal drug products in the development phase, as well as for quality control and bioavailability/bioequivalence studies, regulatory agencies, like the Food and Drug Administration (FDA) and the European Medicines Agency (EMA), have published guidelines and regulations proposing various test methods [8,9,10,11]. Table 1 and Table 2 give a summary of the recommended tests for the different nasal drug products. However, in order to obtain reliable results, the test methods need to be validated, and in this context, it is essential to know the factors that can influence the measurements. In some studies, it could be shown that the spray characteristics can be influenced by the design of the device, by the formulation properties, like viscosity and surface tension, and by the handling of the device, *i.e.*, the actuation parameters [5,12,13,14,15,16,17,18]. Additionally, the selected technique and the set-up of the measurements can also have an effect on the results and have to be considered during method development.

**Table 1:** Tests recommended for the finished drug product specification by the Food and Drug Administration (FDA) and the European Medicines Agency (EMA) (standard quality tests are not listed).

Test	Pressurized metered dose nasal sprays	Nasal powders	Single and multiple use nasal drops	Single and multiple use nasal sprays
Specifications for the drug product				
Pump/valve delivery	yes ^#^			yes ^#^
Delivered dose/content uniformity		yes	yes, for multiple use drops	yes, for multiple use sprays
Dose content uniformity through container life	yes ^#^			yes ^#^
Content uniformity/uniformity of dosage units	no *	no *	yes, for single use drops *	yes, for single use sprays *
Mean delivered dose	yes *	yes *	yes, for multiple use drops *	yes, for multiple use sprays *
Spray pattern	yes ^#^			yes ^#^
Particle/droplet size distribution	yes	yes	no	yes
Particle size distribution of API	yes, for suspensions ^#^			yes, for suspensions ^#^
Microscopic evaluation	yes, for suspensions ^#^			
Particulate matter	yes ^#^			yes ^#^
Microbial limits	yes	yes	yes	yes
Preservative content	no *	no *	yes, if present *	yes, if present *
Preservatives and stabilizing excipients assay				yes ^#^
Sterility	no *	no *	yes, if product is sterile *	yes, if product is sterile *
Net content/minimum fill	yes ^#^			yes ^#^
Number of actuations per container	yes *	yes *		yes, for multiple use sprays *
Weight loss (stability)				yes ^#^
Leachables (stability)	yes ^#^			yes ^#^
Osmolality				yes ^#^
Viscosity				yes ^#^
Appearance and color of content and container closure system	yes ^#^			
Water or moisture content	yes	yes	no	no
Dehydrated alcohol content	yes, if used as a cosolvent ^#^			
Leak rate	yes	no	no	no
Pressure testing	yes, if cosolvent or more than one propellant is used ^#^			

Explanatory note: “yes”, the test is recommended for the particular drug product; “no”, the particular drug product is excluded from the test; blank, no specific details in the guidelines are available; ^#^ FDA only requirement; * EMA only requirement; API, active pharmaceutical ingredient.

This article gives an overview of the regulatory requirements regarding the determination of droplet size distribution (DSD), plume geometry, spray pattern and shot weights. These tests, among others, are required in development and *in vitro* bioequivalence studies, as well as in quality control matters. In addition to the regulatory requirements, analytical challenges and possible influencing factors related to the device, formulation composition and selected method/technique that affect nasal spray characteristics are reviewed. In order to support findings from the literature, studies comprising the determination of DSD, plume geometry, spray pattern and shot weights were performed using model formulations and a standard nasal spray pump.

**Table 2:** Tests recommended for nasal drug product characterization/development studies by the FDA and the EMA.

Test	Pressurized metered dose nasal sprays	Nasal powders	Single and multiple use nasal drops	Single and multiple use nasal sprays
Drug product characterization/development studies				
Physical characterization	yes, for suspensions *	yes *	yes, for suspensions *	yes, for suspensions *
Priming and repriming (in various orientations)	yes	no	no	yes
Plume geometry	yes ^#^			yes ^#^
Microscopic evaluation	yes, for suspensions ^#^			
Effect of resting time	yes ^#^			
Shaking requirements	yes, for suspensions	no	yes, for suspensions	yes, for suspensions
Minimum fill justification	yes *	yes *	yes *	yes *
Extractables/leachables	yes *	no *	yes *	yes *
Performance after temperature cycling	yes	no	no	yes
Effect of environmental moisture	yes *	yes *	no *	no *
Cleaning instructions	yes	yes	yes, for multiple use drops	yes, for multiple use sprays
Device robustness	yes	yes	yes	yes
Profiling of sprays near container exhaustion (tail off characteristics)	yes ^#^			yes ^#^
Delivered dose uniformity through container life	yes *	yes *	yes, for multiple use drops *	yes, for multiple use sprays *
Effect of storage on PSD	yes, for suspensions ^#^			yes, for suspensions ^#^
Particle/droplet size distribution	yes	yes	no	yes, for multiple use sprays
Preservative effectiveness (and sterility maintenance)	no	no	yes, if present	yes, if present
Photostability	yes, if drug is exposed to light ^#^	yes, if drug is exposed to light ^#^	yes, if drug is exposed to light ^#^	yes, if drug is exposed to light ^#^
Actuator/mouthpiece deposition	yes	yes	no	yes *
Determination of appropriate storage conditions	yes ^#^			
Stability of primary (unprotected) package	yes ^#^			yes ^#^
Delivery device development	yes	yes	yes	yes
Microbial challenge	yes ^#^			
Effect of dosing orientation				yes ^#^
In vitro dose proportionality	yes, for suspensions in multiple strengths ^#^			yes, for suspensions in multiple strengths ^#^
Low temperature performance	yes *	no *	no *	no *

Explanatory note: “yes”, the test is recommended for the particular drug product; “no”, the particular drug product is excluded from the test; blank, no specific details in the guidelines are available; ^#^ FDA only requirement; * EMA only requirement; and PSD, particle size distribution.

## 2. Experimental Section

### 2.1. Materials

Mechanical nasal spray pumps delivering 100 μL of formulation per actuation were provided by Aptar (Radolfzell, Germany). Water was used in double-distilled quality (FinnAqua 75, San Asalo-Sohlberg Corp., Helsinki, Finland). Sodium carboxymethyl cellulose (Tylopur C 30 G) was obtained from Clariant (Muttenz, Switzerland) and polysorbate 80 from Uniqema (Snaith, UK).

### 2.2. Model Formulations

The basic formulation was water. For investigating formulation-dependent variables, the viscosity was varied by adding 1%–5% sodium carboxymethyl cellulose (NaCMC) and the surface tension, respectively, by the addition of 0.0001%–0.1% polysorbate 80.

### 2.3. Determination of Viscosity and Surface Tension

Viscosity was measured using a Vibro viscosimeter (A&D Company Ltd., Tokyo, Japan) at room temperature, and the surface tension of the water and model formulations was determined using a plate tensiometer (Processor Tensiometer K 12, Krüss GmbH, Hamburg, Germany).

### 2.4. Determination of Droplet Size Distribution

The droplet size distribution (DSD) was determined by laser diffraction using HELOS with SPRAYER-module and ROTOR, as well as the force and traject actuator, respectively (Sympatec GmbH, Clausthal-Zellerfeld, Germany). The spraying angle was varied between 0° and 90°; the actuation force between 20 and 100 N. The distance to the measuring zone ranged from 3 to 7 cm, and the stroke length was set between 1 and 7 mm. Time-resolved measurements were performed, and data were analyzed according to the Fraunhofer theory. All determinations were performed in triplicate.

### 2.5. Determination of Plume Geometry

For the determination of plume geometry, an Imager E-lite CCD-camera (charge-coupled device camera) and sheet light (LaVision, Göttingen, Germany) were used. The images were corrected for distortion, due to the skewed camera perspective, and plume angle was determined manually using CorelDraw X6 software (Corel, Ottawa, ON, Canada).

### 2.6. Determination of Shot Weights/Validation of Pump Delivery

In order to determine the shot weights, the nasal sprays were filled with 10.0 mL of the respective formulation. The device was actuated with an automated actuator (SPRAYER-module, Sympatec), and after each actuation, the device was weighed on an analytical balance (A 200 S, Sartorius, Göttingen, Germany) to determine the delivered mass. The actuation parameters were set as follows: the actuation force ranged from 40 to 100 N, and the stroke length was varied between 1 and 7 mm.

## 3. Results and Discussion

### 3.1. Droplet Size Distribution

The DSD of a nasal spray is a critical parameter, since it significantly influences the *in vivo* deposition of the drug in the nasal cavity [19]. The droplet size is hereby mainly influenced by the design and handling, e.g., the actuation parameters, of the device, as well as by the formulation, and the prevalent median droplet size is between 30 and 120 μm [20]. If the droplets are too large (>120 μm), deposition takes place mainly in the anterior parts of the nose, and if the droplets are too small (<10 μm), they can possibly be inhaled and reach the lungs [4,20], which should be avoided because of safety reasons.

#### 3.1.1. Regulatory Aspects

In order to determine the DSD of a nasal spray, the FDA and the EMA [8,9,11] recommend making use of laser diffraction, which has already become the standard technique in the industry for droplet and particle size analysis [4]. Laser diffraction is a fast and efficient method that measures the geometric size of droplets and particles in real-time based on two common light scattering principles, which are Mie- or Fraunhofer-theory [19,21]. When determining the DSD, time-resolved measurements should be performed, *i.e.*, the droplet size and obscuration or transmission are recorded at defined time intervals, e.g., every 1 ms, over the entire spray event. On the basis of time history profiles (obscuration/DSD *versus* time), the spray event can then be characterized by three distinct phases: the formation phase, which is indicated by a rapid increase in obscuration and a decrease in droplet size, followed by the fully developed phase, where obscuration and droplet size attain a plateau, and, finally, the dissipation phase, designated by a rapid decrease in obscuration and an increase in droplet size [9,20]. For *in vitro* equivalence purposes, the FDA recommends determining the time history profiles of droplet sizes and obscuration over the complete life of the single spray at two distances ranging from 2 to 7 cm from the nozzle tip, with the two distances separated by 3 cm or more [9]. For new drug applications (NDAs), only one distance within this range is requested [8,21]. In both cases, the data to report should be collected only during the fully developed phase and should comprise the droplet size expressed as *D*_10_, *D*_50_ and *D*_90_, as well as the span defined as (*D*_90_ − *D*_10_)/*D*_50_ as an indicator for the width of the distribution and, for NDAs, additionally, the fraction of droplets smaller than 10 µm [8,9]. It has to be defined and stated by the applicant at which region of the plateau phase the droplet size data were determined. For this, the FDA suggests three different variants of data analysis: “the average of all scans over the entire plateau, the data of a single scan only at the maximum obscuration or the average of a specified range of scans around this obscuration” [9]. Additionally, the FDA recommends using an automated actuator to minimize variability.

In contrast to the FDA, the EMA gives less information about the measurement of the droplet size distribution of nasal sprays in their “guideline on the pharmaceutical quality of inhalation and nasal products”, e.g., how to analyze the data. However, the agency also requests that limits for the median droplet size and the fraction <10 µm should be given [11].

#### 3.1.2. Analytical Aspects

The droplet size distribution can be influenced by various factors, which have to be considered when performing droplet size measurements. Those influencing factors comprise formulation-related properties, like viscosity and surface tension, the design of the device, the actuation parameters, e.g., actuation force, stroke length, actuation velocity, but also method-dependent variables, like the spraying angle or the distance between the nozzle and the laser beam.

##### 3.1.2.1. Formulation Dependent Variables

The formulation of a nasal spray, among other factors, plays an important role in nasal drug delivery. In order to improve nasal drug delivery, some solution-based formulations contain bioadhesive polymers to increase the residence time of the formulation on the mucosa and to control drug absorption. The addition of such polymers also leads to an increase in viscosity, which consequently has an impact on droplet size distribution and drug deposition in the nasal cavity [18]. Harris *et al.* [18] have studied the influence of the addition of methylcellulose to a solution containing desmopressin and noticed an increase in droplet size with an increasing amount of polymer from 53 (water) to 200 µm (0.5% methylcellulose). Dayal *et al.* [14] have investigated the influence of the addition of NaCMC and Carbopol, respectively, on the droplet size distribution of an aqueous nasal spray in comparison to water. For solutions with NaCMC, they observed an increase in droplet size with an increasing amount of polymer for different spray pumps, whereas the *D*_90_ underwent a more significant change than the *D*_10_. Additionally, they could show a linear relationship between the viscosity and the corresponding *D*_50_. For Carbopol solutions, Dayal *et al.* also reported an overall increase in droplet size with an increasing amount of polymer compared to water, but the effect was less pronounced than for the solutions with NaCMC. Moreover, the difference in droplet sizes between the two tested Carbopol solutions was very small. The difference between the two polymers can be explained by their differing rheological behavior: while the carboxymethyl cellulose (CMC) solution behaved like a Newtonian fluid at the low concentration used, the Carbopol solutions exhibited a shear-thinning behavior, and hence, the viscosity is reduced when the liquid is atomized by the spray pump, leading to smaller droplet sizes than initially expected. Pennington *et al.* [22] have also measured the DSD of solutions containing Newtonian viscosity modifiers, namely polyethylene glycol (PEG), propylene glycol (PG) and glycerin in different concentrations. They also discovered that with increasing amount of modifier, the viscosity and the droplet size increases, but they did not detect a linear, but a logarithmic relationship between those parameters. The fact that an increase in viscosity leads to an increase in droplet size was also evaluated statistically using a Box–Behnken experimental design by Guo *et al.* [5]. They also investigated aqueous solutions of NaCMC and showed that viscosity is a statistically significant factor influencing DSD. A Box–Behnken experimental design was also used by Dayal *et al.* [13] in order to evaluate the effect of the surface-active polymers, hydroxyethylcellulose (HEC) and polyethylene-oxide (PEO), in combination with ionic excipients (sodium chloride and calcium chloride) on the viscosity and DSD of nasal sprays. They found that the viscosity of the solutions was increased with an increasing amount of polymer, showing a slight shear thinning behavior, and that the addition of ionic excipients significantly affected the viscosity of the polymer solutions. For HEC and PEO solutions, Dayal *et al.* reported an increase in *D*_50_ in a concentration-dependent manner, with PEO showing larger droplets than HEC, as well as an interaction of polymer and electrolytes, resulting in different DSD, due to changes in viscosity.

**Figure 1:**
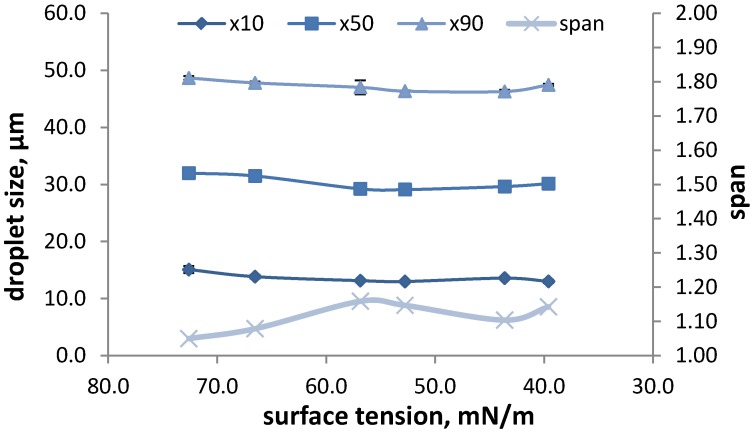
The effect of surface tension on the droplet size distribution (DSD) and span measured at 7 cm from the nozzle and an actuation force of 60 N. Results are presented as the average ± SD of three actuations.

Alongside viscosity modifiers and other excipients nasal spray formulations can also contain surfactants, e.g., to solubilize the active pharmaceutical ingredient (API) or to enhance drug absorption. However, surfactants also have an impact on the surface tension, which can possibly influence DSD. Two groups [5,14] have studied the influence of polysorbate 80 on the droplet size of nasal sprays. Dayal *et al.* showed that an increase in surfactant resulted in a decrease in droplet size, but this effect was very slight. Guo *et al.*, however, could not detect a significant influence of the surfactant concentration on the DSD. In our own studies, we also investigated the influence of polysorbate 80 on DSD. The surfactant was varied between 0.0001% and 0.1%, resulting in surface tensions between 66.5 and 38.4 mN/m. Water showing a surface tension of 72.6 mN/m was also measured as a comparison. The results are shown in Figure 1. There is no significant influence on the *D*_10_, *D*_50_ and *D*_90_, which supports the findings by Guo *et al.*, and only a slight effect on the span was observed, which shows some fluctuations.

Generally, it can be said that the viscosity has a major influence on the DSD, leading to an increase of the droplet size of the nasal sprays, whereas the surface tension only has a minor to no effect on DSD.

##### 3.1.2.2. Device-Dependent Variables

Multi-dose nasal spray pumps, meanwhile, are available from a variety of different manufacturers. Essentially, they are all based on the same principle, *i.e.*, the dose is divided in a metering chamber and forced through a nozzle to disperse the liquid into fine droplets. However, mechanical spray pumps can differ in spray performance, due to modifications of the swirl chamber and inlet channels, altering the dimensions and geometry of the orifice diameter or differences in the pressure that build up in the volume chamber prior to dispensing [23].

Suman *et al.* have compared two different nasal spray pumps regarding droplet size. In one study, they found statistical differences in *D*_50_ values at varied distances between the nozzle and laser beam [24]. In a second study, they compared nasal spray pumps differing in their mechanical operation and could not detect differences in *D*_10_ and *D*_50_ values, but significant differences in *D*_90_ [15]. In this case, the droplet sizes differed by more than 13%. Dayal *et al.* [14] have also evaluated the influence of pump design on the DSD and concluded that the nozzle orifice has an impact on the droplet size that is emitted, since alterations in the diameter, shape and length will affect the compression forces of the liquid, friction and spray velocity. However, in a statistical evaluation, they stated that changes in the formulation have a greater impact on the *D*_50_ than the design of the device.

##### 3.1.2.3. Method- and Actuation-Dependent Variables

The handling of a nasal spray device, *i.e.*, the actuation parameters, like stroke length, actuation force, actuation velocity or hold time, as well as the set-up of the measurements (time point, distance to measuring zone, spraying angle) can significantly influence the DSD and other *in vitro* tests. Generally, correct actuation parameters have to be determined for a selected device [4] and should mimic hand actuation [25].

As described in Section 3.1.1, the FDA suggests determining the DSD during the fully developed phase of the spray event, which leads to the highest degree of reproducibility [26] and most stable droplet size values [27]. Choosing an inaccurate time point can result in an under- or over-estimation of the DSD, and presently, the FDA does not give recommendations on how to determine the stable phase. Guo and Doub [17] conclude that the obscuration thresholds should be defined as close to the maximum obscuration as possible in order to avoid extreme droplets, which are present at the beginning and end of the spray event. They set their lower threshold to 90% of the maximum obscuration in order to define the fully developed phase. Eck *et al.* [26], however, defined the stable phase at 25%–30% absolute obscuration and determined the droplet size at the maximum obscuration. This is only possible when there are no fluctuations present in the time-history plot; otherwise, the data have to be averaged over the entire plateau of the spray [14]. It has also to be taken into account that obscuration values vary with changes in the actuation parameters, the device and the distance to the measuring zone [14,17], and hence, obscuration thresholds have to be defined for every selected device and measurement set-up.

**Figure 2:**
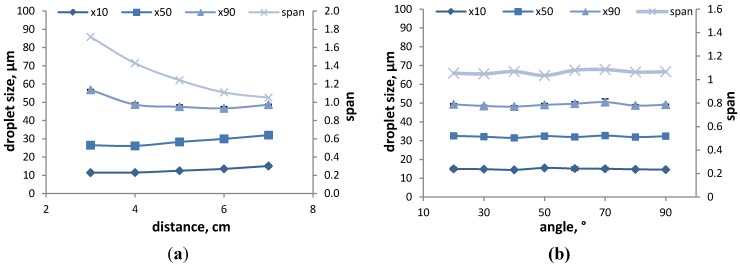
The effect of distance to the measuring zone (**a**) and spraying angle (**b**) on the DSD and span of the model formulation (water) measured at an actuation force of 60 N. The results represent the average ± SD of three actuations.

For the measurement set-up, the FDA requests to select one or two distances for *in vitro* equivalence purposes in the range of 2 to 7 cm (refer to Section 3.1.1). It is known that the distance between the nozzle and the laser beam affects the DSD measurement, due to different settling velocities of the droplets, the plume dynamics and the varied representation of the true DSD in the measurement zone [14,17,26]. In the literature, there are controversial findings regarding the influence of the distance reported. Eck *et al.* [26] determined the DSD of a commercial nasal spray solution at 1, 2.5 and 5 cm and found that an increase in distance led to a decrease in *D*_10_, *D*_50_ and *D*_90_ values for the stable phase. In a study by Guo and Doub using water as a model formulation [17], the actuation distance was varied between 1 and 9 cm, and they also observed significant differences in *D*_50_. In the range of 1 to 3 cm, the *D*_50_ decreased, showing the smallest value at 3 cm, and then increased with increasing distance, showing the largest overall value at 9 cm. In our own studies, we have varied the distance to the measuring zone between 3 and 7 cm and observed an increase in *D*_10_ and *D*_50_, as well as a decrease in span with increasing actuation distance (Figure 2a) using water as a model formulation. The reason might be that the determination at higher distances provides more time for the plume to develop and, hence, smaller droplets are able to coalesce and form larger droplets, which leads to an overall increase in the droplet size. Dayal *et al.* [14] have performed measurements at a distance of 1.5, 3 and 6 cm using commercial nasal sprays and reported a decrease in DSD with increasing distance to the measuring zone. This was explained by the assumption that with greater distances, part of the droplets escape from the measuring zone, resulting in a larger percentage of droplets, and, consequently, the measured data is not representative of the entire DSD of the spray. Therefore, Dayal *et al.* suggest that actuation at a short distance may provide a better representation of DSD. However, measurements at a short distance may lead to multiple scattering, due to the high density of droplets in the measuring zone, which can result in an underestimation of droplet size and, consequently, a distance has to be chosen based on the obscuration levels that reduce multiple scattering events [14].

A second set-up parameter for DSD measurements besides the actuation distance is the spraying angle. Most routine measurements are performed at a fixed angle, which has to be defined, but in order to determine, e.g., a position (in)dependency of DSD, determinations should be performed at a variety of angles. In this study, the position angle of the nasal spray was varied between 0° (horizontal set-up) and 90° (upright position) to determine an adequate angle for routine measurements and to evaluate the position dependency of the DSD. For the 0° and 10° angles, no data could be obtained, since no dose was released from the device after actuation. However, the results for measurements at angles between 20° and 90° show that there is no significant change in DSD and span with varying spraying angles (Figure 2b). Consequently, the DSD generated by this particular device is not dependent on the position of the nasal spray once a dose is metered. For routine measurements, an angle of 70°–80° is suggested, since obscuration values are more stable in this region than for smaller angles (data not shown), and this range comes close to in-use conditions when patients administer a nasal spray.

As mentioned before, actuation parameters can also influence DSD and other spray characteristics. Consequently, it is crucial to know their influence and to select them carefully for every device considering the age (pediatric, adult, geriatric settings) and gender of the target group [4,25]. For *in vitro* tests, automated actuation systems should be used in order to control actuation, to increase the reproducibility of measurements and to minimize operator bias. More information about automated actuation systems can be found by Guo and Doub [17] and Dayal *et al.* [14]. In a study, Guo and Doub have evaluated the influence of several actuation parameters on the DSD and found out that the hold time, return stroke velocity and acceleration do not influence nasal spray characteristics. Therefore, these parameters are not discussed here any further, and the focus is put on actuation velocity, stroke length and actuation force.

The actuation velocity has a large impact on DSD. Guo and Doub found that with increasing velocity, the *D*_50_ decreases, which is significant for the lower velocity settings, but in the region of optimal velocities, the droplet size tends to stay stable. These findings are supported by a study by Guo *et al.*, who found in their experimental design study that actuation velocity is a significant factor and that an increase in velocity leads to a decrease in *D*_10_, *D*_50_ and *D*_90_. However, this factor shows interactions with the amount of viscosity modifier added, which makes interpretation of the responses more complicated. In addition, Kippax *et al.* [28] concluded that an increase in actuation velocity (40–100 mm/s) leads to a decrease in *D*_50_, and this was also true for solutions containing different amounts of polyvinylpyrrolidone to modify the viscosity.

**Figure 3:**
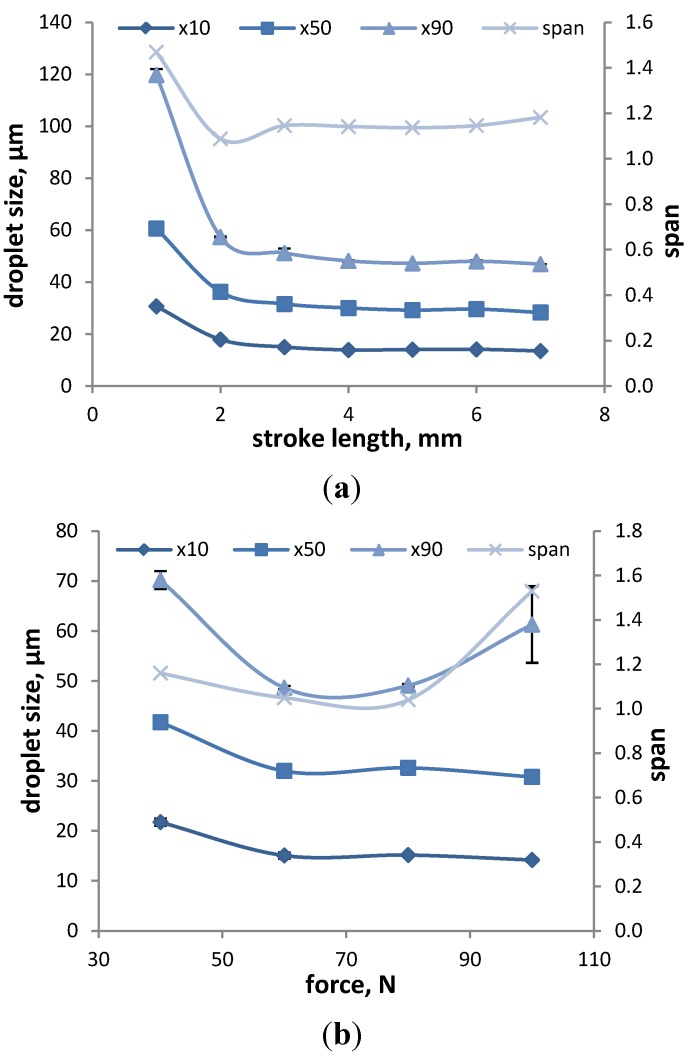
The effect of stroke length (**a**) and actuation force (**b**) on the DSD and span of the model formulation (water) measured at 7 cm from the nozzle. The results represent the average ± SD of three measurements.

Guo *et al.* have also investigated the influence of stroke length on the DSD and concluded that this factor does not significantly influence the droplet size. Guo and Doub showed that stroke length has only a slight effect on DSD in the normal actuation range, but at lower settings, the *D*_50_ decreases with increasing stroke length. This dependency can also be supported by our studies (Figure 3a), where the stroke length was varied between 1 and 7 mm. For stroke lengths of 1–3 mm, there is a dramatic decrease in *D*_10_, *D*_50_ and *D*_90_, as well as the span with increasing stroke length, but for the range of 4–7 mm, the DSD and span reach a plateau and remain stable. The reason is that with the lower settings, the device is not actuated properly, which can be asserted by the fact that the emitted mass does not reach the target value (refer to Section 3.3.2), and consequently, there is not enough energy provided to disperse the liquid into fine droplets. However, once the device is actuated with an optimal stroke length, the droplets size remains constant.

The impact of actuation force on DSD was investigated by Dayal *et al.* [14], and in their study, they could show that an increase in actuation force (3 to 7 kg) led to a decrease in *D*_50_ values by 37%. In our study, the actuation force ranged from 20 to 100 N (1 N = mass (kg) × 9.81 m/s^2^), and the results for DSD and span are shown in Figure 3b. Twenty newtons were not sufficient to actuate the device, and consequently, no data could be gathered for this adjustment. The other results show that with increasing actuation force, the *D*_10_ and *D*_50_ values tend to decrease and that the *D*_90_ values show a high variability for measurements at 100 N. Generally, the adjustment of the actuation force should represent the force employed by the relevant patient group [14] for the particular device to be tested. Dayal *et al.* determined an actuation force of 4.5 kg for their device and Doughty *et al.* [29] determined a mean actuation force of 5.82 kg for adults and 3.37 kg for children. Therefore, an actuation force of 100 N is too high and not feasible for DSD measurements, in contrast to actuation forces between 40 and 60 N.

### 3.2. Plume Geometry and Spray Pattern

Plume geometry testing requires images taken from a sideward view of the emitted spray parallel to the axis of the plume, whereas for the evaluation of the spray pattern, an image of an axial cross-section of the plume at a defined distance to the nozzle is compulsory. These two characteristics can be influenced by, e.g., “the size and shape of the nozzle, the design of the pump, the size of the metering camber and the characteristics of the formulation” [8].

#### 3.2.1. Regulatory Aspects

In order to determine the plume geometry, the FDA recommends to either make use of high-speed photography or a laser light sheet and a high speed digital camera. The quantification can be performed manually or by automated image analysis. The evaluation of plume geometry and spray pattern is only requested by the FDA. The EMA has not included these or similar tests into their guidelines. With the selected technique, it should be possible to monitor the development of the plume to define the shape, *i.e.*, two side views at 90° to each other and relative to the axis of the plume should be employed [8]. However, for *in vitro* equivalence testing, one side view is sufficient [9]. The evaluation of plume geometry can be performed manually or by automated image analysis and should include plume angle, width and height at a single delay time during the fully developed phase of the spray event. The plume should be still in contact with the nozzle of the device, and the plume angle “would be based on the conical region of the plume extending from a vertex that occurs near or at the actuator tip” [9]. The FDA further recommends determining the plume width at the greater distance of the two distances chosen for evaluating the spray pattern. Accordingly, the data of the plume width are complimentary to the spray pattern data. In contrast to spray pattern analysis, which is mandatory for the release of the drug product in terms of quality control, the determination of the plume geometry is a part of drug product characterization studies and does not have to be tested on a routine basis thereafter.

The spray pattern of a nasal spray can be characterized using either impaction or non-impaction systems. For impaction systems, an adequate collection surface, e.g., a plate used for thin layer chromatography (TLC-plate), can be employed, and the visualization technique should be specific for the drug substance, if possible. The characterization of the spray pattern can also be performed by manual or automated image analysis. When the spray pattern is evaluated manually, the approximate center of mass (COM) should be identified, and the maximum diameter (*D*_max_) and minimum diameter (*D*_min_) should be drawn through this center to determine the size of the pattern. Additionally, the ovality ratio, defined as *D*_max_/*D*_min_, can be determined as the control of the shape of the pattern. Automated image analysis software can also define the perimeter of the true shape, as well as the center of gravity (COG) and is expected to increase the objectivity of spray pattern analysis. For non-impaction analysis systems based on a laser light sheet and a high-speed digital camera, which allow the visualization of a cross-section of the plume, can be used. The automated image analysis should also include the perimeter of the true shape and the determination of COG, COM, *D*_min_, *D*_max_, as well as the area within the perimeter. For non-impaction systems, the spray pattern should be determined based on single actuations, whereas using impaction systems, the measurement can be based on multiple actuations, but if possible, should be based on only one actuation. Spray pattern measurements should be performed at two distances from the actuator tip, and the selected distances should be at least 3 cm apart within the range of 3 to 7 cm.

#### 3.2.2. Analytical Aspects

Kulkarni and Shaw [4] have concluded that a uniform circular plume with an ovality ratio close to one can be considered ideal. In order to determine the plume geometry and spray pattern, various factors have to be considered, influencing the measurements. One of them is the selection of the adequate measuring technique. For the determination of plume geometry, Farina [25] suggests using a laser sheet and a high-speed digital camera, since flash photography techniques are difficult to validate and most often comprise subjective analysis. The drawback of analyst bias and subjectivity is also related to the impaction systems, *i.e.*, the TLC-plate tests, when performing spray pattern measurements. Additionally, these tests are very time consuming and require a permanent storage of the TLC-plates [17]. Hence, Farina [25] recommends using non-impaction systems to increase the objectivity of data analysis.

##### 3.2.2.1. Formulation-Dependent Variables

**Figure 4:**
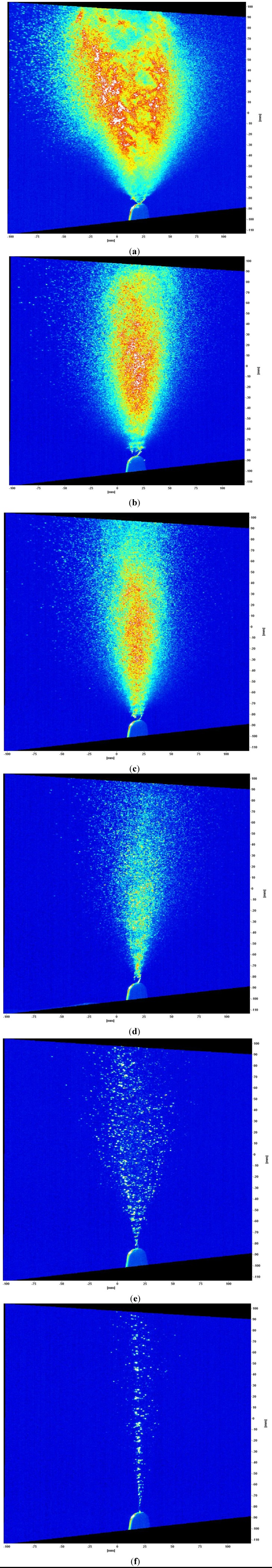
The influence of viscosity on the plume geometry. (**a**) Water; (**b**) 1% sodium carboxymethyl cellulose (NaCMC); (**c**) 2% NaCMC; (**d**) 3% NaCMC; (**e**) 4% NaCMC; and (**f**) 5% NaCMC; the device was actuated manually.

**Figure 5:**
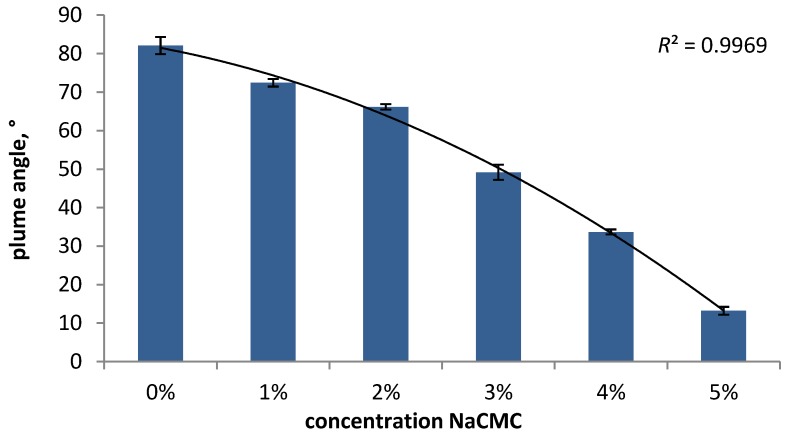
The influence of viscosity on the plume angle.

The major formulation-dependent variables that can influence plume geometry and spray pattern are the viscosity and surface tension. Guo *et al.* [30] have investigated model formulations containing microcrystalline cellulose/NaCMC (0.25%–2.0%) using open-flash digital photography and the TLC-plate method. The results show that an increase in viscosity leads to a decrease in plume angle (68.7° to 44.9°) and an increase in plume width and height. The spray pattern analysis demonstrates that with increasing viscosity, *D*_min_ decreases clearly, whereas in *D*_max_, there is only a difference between the formulations containing 0.25% and 0.5% of the viscosity modifier. However, no explicit changes in the ovality ratio could be observed. Guo *et al.* [5] have evaluated different solutions containing NaCMC in an experimental design study using a non-impaction automated image analysis system and confirmed that the viscosity has a significant influence on the plume geometry and spray pattern. The optimized models show that with increasing concentration of polymer, the spray angle, as well as the plume width and the spray pattern area are reduced. Dayal *et al.* [14] have also investigated model formulations containing NaCMC, as well as Carbopol using the TLC-plate method and described also a reduction of the spray surface area and changes in the spray shape with increasing viscosity. However, the changes in the spray pattern are more evident for NaCMC solutions, resulting in a power-law relationship between spray surface area and viscosity, whereas for Carbopol formulations, they could not detect a correlation. The reason for the diverse results is based on the different rheological behaviors of the two polymers, which is discussed in Section 3.1.2. The influence of the rheological behavior and viscosity of different Newtonian fluids (polyethylene glycol, propylene glycol and glycerin) on the spray pattern was investigated by Pennington *et al.* [22] using a non-impaction technique. They also concluded that an increase in viscosity leads to a decrease in spray area, described by an exponential relationship, which was observed for all three investigated substances. In our studies, we have also investigated the influence of viscosity on the plume geometry. The results are shown in Figure 4 and support the findings of the previous studies described above, *i.e.*, a decrease in plume angle with increasing viscosity. The image of water (Figure 4a) shows a wide angled plume, with the liquid dispersed into fine droplets, whereas for a solution containing 5% NaCMC, the plume is jet-like, with obvious larger droplets, which was also confirmed by DSD measurements (data not shown). The impression that the plume geometry is changed by modifications in viscosity can also be supported by the determination of the plume angle (Figure 5). The plume angles decrease from 82.1° (water) to 13.2° (5% NaCMC) following a polynomial relationship (*R*^2^ = 0.9969).

In contrast to viscosity the surface tension of the formulation does not have a significant influence on the plume geometry and spray pattern [5].

##### 3.2.2.2. Device-Dependent Variables

The design of the device can also affect the plume geometry and spray pattern. The shape of the spray pattern, e.g., can be round or oval, as well as star-shaped or even more complex for the same formulation and distance between the nozzle and the TLC-plate using different devices [14]. Additionally, differing intensities within the patterns account for asymmetrical spray characteristics [14], which are also influenced by the device. In two studies, Suman *et al.* [15,24] evaluated nasal spray pump performances and found statistical differences between the devices regarding plume geometry and spray pattern. The tests revealed differences in spray angle, plume width and length (plume geometry), as well as in *D*_min_, *D*_max_ and the ovality ratio (spray pattern).

##### 3.2.2.3. Method- and Actuation-Dependent Variables

As for other spray characteristics, the plume geometry and spray pattern can be influenced by the set-up of the measurement, as well as by the actuation parameters. The determination of the spray pattern can be assessed with a fully automated procedure, which increases the objectivity of the analysis, while plume geometry measurements can only be performed on a semi-automated basis, since the analyst has to choose the image frame at the fully developed phase of the spray event manually [17].

In the determination of the spray pattern, the distance between the nozzle and the TLC-plate and laser sheet, respectively, has an influence on the test results. Suman *et al.* [24] could not detect differences between two nasal spray pumps when measurements were performed at 1 cm. When the tests were performed at 2.5 and 5 cm, however, the devices showed significant differences in *D*_max_ and *D*_min_. Guo and Doub [17] have performed their measurements at two distances (3 and 6 cm), and their results revealed that with increasing distance, there is an increase in the spray pattern area for all tested actuation parameters, but distance does not have a distinct impact on the ovality ratio of the spray pattern.

In the literature, there is no evidence that actuation force has an effect on the plume and spray pattern characteristics. However, it can be assumed, deducing from the DSD measurements, that the plume angle will decrease with decreasing actuation force, since the available energy for the dispersion of the solution will also decrease, leading to larger droplets and a narrower spray plume.

The most dominant actuation parameter in this context is the actuation velocity. The plume width and plume angle, as well as the spray pattern area increase with increasing actuation velocity [5,17]. Additionally, an increase in actuation velocity also leads to a slight decrease in spray pattern ovality.

In the above-mentioned studies, Guo *et al.* [5,17] could also show that stroke length only has a minimal impact on the plume characteristics. An increase in stroke length leads to a slight increase in plume width and angle, as well as the spray pattern area, but the effects are not significant for the normal actuation range.

In summary, from the available actuation parameter adjustments, only the actuation velocity has a significant influence on the plume characteristics.

### 3.3. Shot Weights/Pump Delivery

The determination of shot weights serves to check the functionality of the valve and to assess pump-to-pump reproducibility in terms of drug product performance to assure reproducible and precise dosing [8,21]. In order to determine shot weights for a nasal spray, the device should be weighed prior and after each actuation on an analytical balance to assess the emitted mass.

#### 3.3.1. Regulatory Aspects

Generally, the determination of shot weights is an FDA-only requirement. The EMA requests in this context the assessment of delivered dose uniformity (through container life) and the statement of the mean delivered dose, whereas the FDA requests the assessment of pump delivery, as well as the determination of spray content uniformity (SCU). However, the EMA states that for solution formulations, the use of the uniformity of weight per actuation in place of SCU may be acceptable if appropriate justification is provided. For example, during development studies for Investigational New Drug Applications (IND), shot weights may serve as a surrogate for SCU, but also only for solution sprays and not for suspension formulations [7].

Generally, the correct performance of the valve should be ensured primarily by the pump manufacturer, who is, in most cases, responsible for the assembly of the nasal spray pump [21]. However, the FDA recommends verifying the pump delivery. For this, the FDA proposes that the “acceptance criteria should control the weight of the individual sprays to within 15% of the target weight and their mean weight to within 10% of the target weight” [8].

#### 3.3.2. Analytical Aspects

The pump delivery of a nasal spray can be influenced by several factors, including viscosity and surface tension, as well as the actuation parameters. However, in the literature, there are only a few studies published dealing with the investigation of factors influencing shot weights.

##### 3.3.2.1. Formulation-Dependent Variables

The viscosity and surface tension of a nasal spray formulation can influence DSD, spray pattern and plume geometry (refer to Section 3.1.2 and Section 3.2.2). Harris *et al.* [18] and Guo *et al.* [5] have also investigated the influence of these formulation variables on pump delivery. Using different solutions containing desmopressin and methylcellulose (0%, 0.25% and 0.5%), Harris *et al.* could show that the viscosity does not have an influence on the shot weights and dosing accuracy, respectively. Guo *et al.* have evaluated different placebo formulations, which contained NaCMC and polysorbate 80 to modify the viscosity and surface tension, in an experimental design study and found out that the concentration of polymer and surfactant have a significant influence on the shot weight model. However, the effect is very slight.

##### 3.3.2.2. Device-Dependent Variables

Basically, the dimension of the metering chamber in the pump determines the volume and the respective mass that is delivered from the device [23]. Typically, nasal spray pumps deliver 100 μL of formulation per spray, but there is a wide range of dosage volumes available (25 to 200 µL) in the market [31].

When the dimensions of the metering chamber are changed, then the emitted mass will be influenced, but there are no differences to be expected between spray pumps of different suppliers when the same metering volume is claimed. Suman *et al.* [24], e.g., have compared the delivered doses between two different nasal spray pumps at the beginning and end of their use cycles and did not detect any statistical differences.

##### 3.3.2.3. Method- and Actuation-Dependent Variables

The handling of the device can also influence the pump delivery. Guo *et al.* found out that besides the concentration of the viscosity modifier and surfactant, the shot weight is dominantly and significantly influenced by the stroke length, since it affects the volume of the formulation, which is pulled into the metering chamber of the valve. Guo and Doub [17] also concluded that stroke length is the dominating factor in the determination of shot weights. In this study, the shot weight increased with increasing stroke length before reaching a plateau at the target delivered mass of 100 mg. For the device used, the stroke length had to be at least 3.8 mm in order to actuate the device properly and to deliver the correct dose; otherwise, less than 90% will be emitted. For actuation velocity and acceleration, Guo and Doub could not detect a significant influence; only the results for very slow actuation accelerations show a high variability. Otherwise, the effects, if any, are very small.

**Figure 6:**
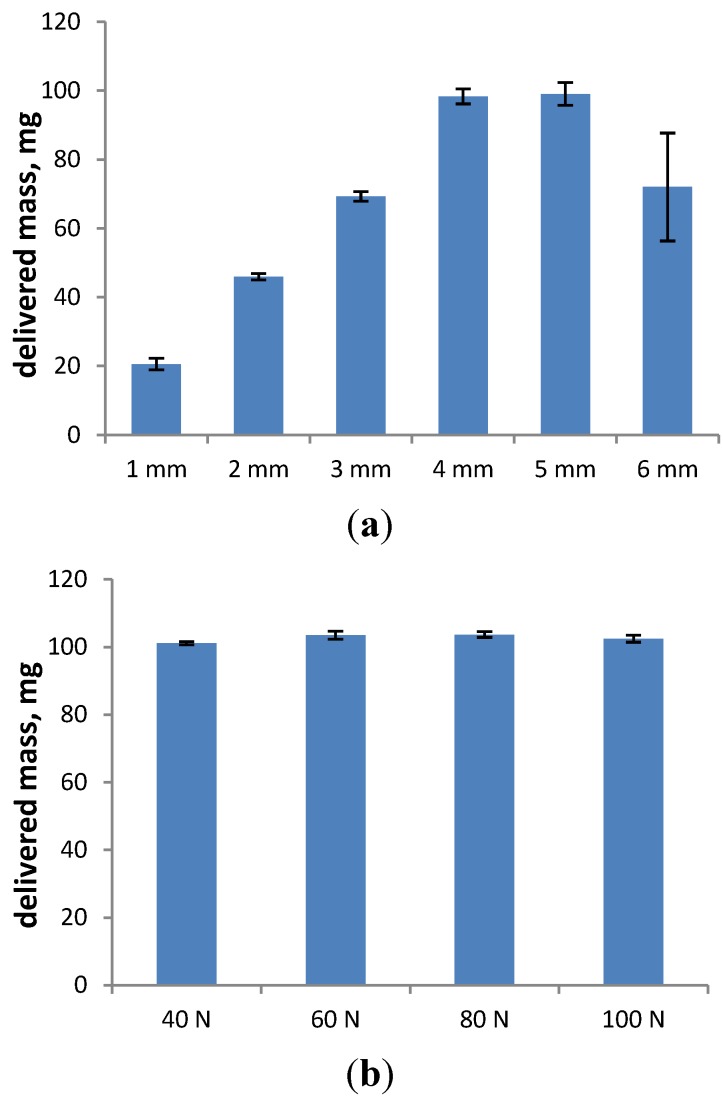
The effect of stroke length varied between 1 and 6 mm (**a**) and actuation force varied between 40 and 100 N; and (**b**) on shot weights. Results represent the average ± SD of at least 90 actuations.

In our studies, we have evaluated the influence of stroke length and actuation force on the pump delivery and mean delivered mass. The results for stroke length are in agreement with the findings by Guo and Doub, *i.e.*, the delivered mass increases with increasing stroke length and stays stable, as well as reaches the target value of 100 mg at the optimal stroke length, which is 4–5 mm for this particular device (Figure 6a). The delivered mass is reproducible for stroke length settings between 1 and 5 mm; no mass is outside the limit of ±15% of the average value (data not shown). However, the target value is only reached at 4 and 5 mm. A stroke length of 6 mm leads to an unreproducible dosing (data not shown) and a mean delivered dose of only 70 mg, showing high variability. In this case, the stroke length was chosen as too high, which led to an “overactuation” of the device, which compromised the functionality of the valve. Consequently, it is crucial to set the stroke length within the optimal range to assure a precise dosing and to avoid damage to the valve.

In contrast to the stroke length, the actuation force (20–100 N) does not have a significant influence on the shot weight (Figure 6b). Twenty newtons were not sufficient to actuate the device, which was known already from the DSD measurements. However, for actuation forces ranging from 40 to 100 N, the target mass of 100 mg is emitted from the device, and the delivered masses are reproducible with no mass outside the limit of ±15% of the target value (data not shown).

## 4. Conclusions

In this study, regulatory requirements and analytical challenges related to the characterization of nasal drug products with the focus on DSD, spray pattern, plume geometry and valve delivery were reviewed, and the findings were supported by our own studies. The FDA and EMA have proposed test methods in different guidelines, but there is a lack of details on how to conduct the studies and what to consider in terms of factors influencing spray characteristics. The results show that nasal spray characteristics can be influenced significantly by various factors, including the formulation, the selected device, as well as the actuation parameters.

Regarding formulation-dependent variables, changes in surface tension have only minimal to no effect on nasal spray characteristics. However, the viscosity of a formulation significantly influences the DSD, plume geometry and spray pattern. Consequently, this impact has to be considered in formulation development, where viscosity modifiers are often used to increase the suspension stability or to prolong the retention time of the formulation in the nasal cavity.

A nasal drug product does not only comprise the formulation, but also the delivery device, which also affects the performance of the spray. The findings show that different device designs and set-ups lead to diverse results in DSD, plume geometry and spray pattern, and hence, the device development and selection, respectively, should be done simultaneously with or even prior to formulation development and the realization of development studies.

Besides the nasal drug product itself, the selection of the test method and the methodology, as well as the adjustment of actuation parameters can influence the test results. Plume geometry and spray pattern can be assessed by means of automated analysis, as well as manually. The latter, however, is designated by a high degree of subjectivity and operator bias. Consequently, automated analysis will lead to more reproducible results. For the determination of the spray pattern and DSD, the distance between the nozzle and the laser sheet/TLC-plate and the measuring zone of the laser diffractometer, respectively, has also an impact on the results and has to be considered in method development. Another impact on nasal spray characteristics also is the adjustment of actuation parameters, and this applies to all test methods containing actuation events. Therefore, the FDA recommends using an automated actuator. Among all the actuation parameters, stroke length, actuation force and actuation velocity have the largest effects and influence shot weights, DSD, plume geometry and spray pattern. Consequently, these parameters have to be chosen carefully and should mimic hand actuation considering the target population group.

Generally, it can be concluded that the characterization of nasal drug products holds various analytical challenges, and in order to perform reliable and reproducible measurements to assure the quality of nasal drug products, critical parameters have to be identified and evaluated.
